# Transcranial Magnetic Stimulation to Address Mild Cognitive Impairment in the Elderly: A Randomized Controlled Study

**DOI:** 10.1155/2015/287843

**Published:** 2015-06-16

**Authors:** Hellen Livia Drumond Marra, Martin Luiz Myczkowski, Cláudia Maia Memória, Débora Arnaut, Philip Leite Ribeiro, Carlos Gustavo Sardinha Mansur, Rodrigo Lancelote Alberto, Bianca Boura Bellini, Adriano Alves Fernandes da Silva, Gabriel Tortella, Daniel Ciampi de Andrade, Manoel Jacobsen Teixeira, Orestes Vicente Forlenza, Marco Antonio Marcolin

**Affiliations:** ^1^Transcranial Magnetic Stimulation Laboratory, Institute of Psychiatry, Faculty of Medicine, University of São Paulo, Rua Dr. Ovidio Pires de Campos 785, 05402-010 São Paulo, SP, Brazil; ^2^Laboratory of Neuroscience (LIM 27), Department and Institute of Psychiatry, Faculty of Medicine, University of São Paulo, Rua Dr. Ovidio Pires de Campos 785, 05402-010 São Paulo, SP, Brazil; ^3^Division of Neurological Surgery, Pain Center, Department of Neurology, University of São Paulo, Avenida Dr. Enéas de Carvalho Aguiar 255, Sala 5084, Cerqueira César, 05403-900 São Paulo, SP, Brazil

## Abstract

Transcranial magnetic stimulation (TMS) is a noninvasive brain stimulation technique with potential to improve memory. Mild cognitive impairment (MCI), which still lacks a specific therapy, is a clinical syndrome associated with increased risk of dementia. This study aims to assess the effects of high-frequency repetitive TMS (HF rTMS) on everyday memory of the elderly with MCI. We conducted a double-blinded randomized sham-controlled trial using rTMS over the left dorsolateral prefrontal cortex (DLPFC). Thirty-four elderly outpatients meeting Petersen's MCI criteria were randomly assigned to receive 10 sessions of either active TMS or sham, 10 Hz rTMS at 110% of motor threshold, 2,000 pulses per session. Neuropsychological assessment at baseline, after the last session (10th) and at one-month follow-up, was applied. ANOVA on the primary efficacy measure, the Rivermead Behavioural Memory Test, revealed a significant group-by-time interaction (*p* = 0.05), favoring the active group. The improvement was kept after one month. Other neuropsychological tests were heterogeneous. rTMS at 10 Hz enhanced everyday memory in elderly with MCI after 10 sessions. These findings suggest that rTMS might be effective as a therapy for MCI and probably a tool to delay deterioration.

## 1. Introduction

Mild cognitive impairment (MCI) is an intermediary status between normal aging and very early dementia [1], wherein individuals have subjective cognitive deficits and objective memory impairment, without affecting their daily activities [2].

MCI is not necessarily a prodrome of Alzheimer's disease (AD), although evidence suggests that patients with the amnesic subtype (a-MCI) are likely to progress to AD [1, 3–7]. Episodic memory decline is the most frequent impairment in patients who will progress to AD (MCI due to AD) [8, 9]. Patients with MCI typically show impairment in delayed recall [10]. A combination of multivariate episodic memory tests increases the prediction of AD converters and identifies the profile associated with each MCI subtype [11]. Likewise, everyday memory, which includes episodic memory, is impaired [12]. Difficulties in episodic memory are common in healthy aging [13], and studies revealed that the Rivermead Behavioural Memory Test (RBMT), a brief battery test for everyday memory, is able of differentiate between individuals with MCI, AD, and healthy controls [12, 14].

Even though some older adults perform as well as young adults [15], memory processing declines with senescence, particularly in episodic memory tasks, which involve encoding and retrieval of information. Episodic memory processes is dependent on the integrity of the medial temporal lobe and the interaction with lateral prefrontal cortex (PFC) [13, 16]. The posterior parietal cortex is also involved in this network [3, 4, 17]. Imaging studies have evidenced that neural activity reductions occur primarily in the left PFC and temporooccipital regions during encoding, and right PFC was important for retrieval [18–21]. This rationale is clinically consistent with the HERA (hemispheric encoding/retrieval asymmetry) model, which predicts that the younger adults' left PFC specializes in encoding and the right PFC specializes in retrieval [15, 18, 19, 21]. In normal aging, PFC activation tends to be less asymmetric during memory tasks, as indicated by the HAROLD (hemispheric asymmetry reduction in older adults) model. Cabeza et al. [15] compared PFC activity in younger adults and in low-/high-performing older adults during memory tasks using PET and fMRI. The results suggest that low-performing older adults recruited a similar network as young adults but used it inefficiently, whereas high-performing older adults counteracted age-related neural decline through a plastic reorganization of neurocognitive networks.

Such cognitive deficits, even mild, cause great distress to the elderly with MCI, who feel that their autonomy, independence, and ability to lead high-quality lives are negatively affected. These impairments are often considered the most debilitating aspect of aging [22].

Transcranial magnetic stimulation (TMS) emerges as a therapeutic tool with clinical benefits in neurological and psychiatric diseases. The method is based on generating a rapidly variable magnetic field over the scalp in awake subjects, which induces a transitory electric current in the cortical surface and modulating neuronal function directly underneath the coil, and in connected brain regions [23–25]. Repetitive TMS (rTMS) at low frequencies (<1 Hz) reduce cortical excitability, whereas high-frequency rTMS (>1 Hz) facilitates neuronal excitability [26, 27].

Thereby, TMS fulfills an important contribution for studying mechanisms of cognitive function and behavioral plasticity in the human brain [28]. As rTMS can interfere transiently with cortical processing [29], change in behavioral and cognitive performances occurs conversely. Repetitive TMS (rTMS) promote modulation of cortical circuits by inducing changes in synaptic plasticity and reorganization of the cortex, modulating neuronal activity beyond the stimulation period [30–32]. The after-effects of repeated sessions may outlast for days and even weeks [4, 33]. Evidence suggests that off-line rTMS might outlast the stimulation period by synaptic LTP and LTD mechanisms [34–36], even at brain sites distant from those stimulated [18, 37].

HF rTMS induces upregulation of N-methyl-D-aspartate (NMDA) receptor activity and increases gamma-aminobutyric acid (GABA) mediated inhibition [38, 39]. rTMS might reach other neuronal processes, such as genetic and protein regulation, and circuit-level patterns, such as network oscillations [40] and changes in neural signaling by triggering the activation of neuromodulators, such as acetylcholine, dopamine, norepinephrine, and serotonin [41]. Moreover, rTMS also leads to nonneuronal processes, such as changes in blood flow [40, 42]. Brain derived neurotrophic factor (BDNF) is part of the neural signals for synaptic plasticity [34, 43]. It is, however, unclear by which mechanism rTMS induces lasting effects on the brain. Nevertheless, such effects are often described as LTD- or LTP-like, respectively, long-term depression and potentiation [30].

With regard to TMS and memory studies, Turriziani et al. [18] reported improvement in recognition memory (verbal and nonverbal) performance after online LF rTMS over right DLPFC of healthy and MCI individuals. Manenti et al. [19] studied the effect of online HF rTMS (20 Hz, 90% MT) during encoding or retrieval of associated and nonassociated word pairs. A predominance of left DLPFC over right DLPFC was observed in the low-performing elderly. The same research group [44] conducted a trial with young subjects, using online HF rTMS (10 Hz, 90% MT) during retrieval phase of a face-naming task (episodic memory retrieval). The results suggest a recruitment of left DLPFC during retrieval without using retrieval strategies, whereas there is a shift to the right DLPFC if retrieval strategies were needed. Solé-Padullés et al. [45] combined functional magnetic resonance imaging (MRI) and off-line HF rTMS (5 Hz, 80% MT) of the left and right DLPFC before memory tasks, improving learning of face-name associations in the elderly with memory dysfunction, with increased metabolic activation of the right DLPFC. However, an angled active coil was used in the sham condition [46, 47], and a double-cone coil was used. Rossi et al. [48] compared the effect of online HF rTMS to the right and left DLPFC (20 Hz, 90% MT) on visuospatial recognition memory of subjects <45 and >50 years old. They reported a greater interference of rTMS to the right DLPFC compared to to the left DLPFC, in younger subjects. This asymmetry is progressively vanished as the age increases. The bilateral interference effects found in the older group corroborates this reasoning and HAROLD model, which the neural retrieval correlates modify along aging as a compensatory functioning of the DLPFC in elders for episodic memory performance.

A recent article [34] reviewed studies on TMS as diagnostic and as therapeutic tool in patients with MCI and AD, suggesting that rTMS can improve or restore several impaired cognitive functions in AD and MCI.

Manenti et al. [49] conducted a systematic review on studies of TMS and episodic memory addressing young and elderly adults and subjects with memory dysfunction. They report that, despite numerous studies on the role of the DLPFC in episodic memory, there are many studies also demonstrating the involvement of a more distributed neural network, sustaining this function involving the temporal lobes and parietal cortices. For example, Cotelli et al. [3], in a single-case report, applied sessions of HF rTMS (20 Hz, 100% MT) to the left parietal cortex of one male patient with a-MCI, in 10 consecutive days. The observed improvement on association memory tasks persisted significantly for 24 weeks after stimulation.

Finally, [50] conducted a systematic review on cognitive effects of HF rTMS studies and its potential long-term effects. The authors included only off-line rTMS studies using more than a single rTMS session. Baseline subjects diagnoses addressed young and older subjects, clinical (neurologic/psychiatric) or not. They verified that HF rTMS (10–20 Hz) is most likely to cause significant cognitive improvement when applied over the left DLPFC, within a range of 10–15 successive sessions and an individual 80–110% MT.

In the present study, we used several neuropsychological measures, including a very sensitive measure of everyday memory (RBMT). We firstly aimed to investigate whether HF rTMS over the left DLPFC improve everyday memory of elderly patients with MCI and, secondly, to evaluate the effects of rTMS in executive functions. We have chosen the left DLPFC as the target area based on previous rTMS and functional neuroimaging studies of memory in healthy and in MCI patients. To date, there has been no randomized controlled double-blind study in this population.

## 2. Materials and Methods

### 2.1. Inclusion Criteria

Thirty-four elderly subjects, both sexes, age ranging between 60 and 74 years, with education level ≥ 4 years, meeting clinical/neuropsychological criteria for MCI for at least one year, were recruited from the community, through media advertisements, between October 2010 and June 2011.

The study protocol was approved by the Local Ethics Committee and all subjects signed informed-consent forms before enrolling in the trial and registering at Clinicaltrials.gov NCT01292382.

#### 2.1.1. Screening Tests: Part I

In the first step of the screening (part I), we used the Montreal Cognitive Assessment test (MoCA test) [51], the Clinical Dementia Rating (CDR) Scale [28], the 15-item Geriatric Dementia Scale (GDS-15) [52], the 17-item Hamilton Depression (HAMD-17) Scale [53], and the14-item Hamilton Anxiety (HAMA-14) Scale [54]. GDS-15 is a diagnostic assessment and evaluates depressive symptoms in the elderly. The HAMD-17 scale is not a diagnostic instrument but quantifies the severity of depression, comprising somatic and psychological parameters, and allows a follow-up of the patient. We use the two scales for screening due to their different approaches of the depressive disorder, often underestimated in a geriatric clinical evaluation. The respective cut-off points for the tests and scales are in Table 1.

#### 2.1.2. Screening Tests: Part II

The second step of the screening (part II) included lab tests, cerebral MRI scan, and neuropsychological evaluation.

*(1) Lab Tests*. Lab tests were performed for clinical screening in order to detect and exclude clinical secondary causes of dementia or cognitive deficits, such as hypothyroidism, SIDA, vitamin B12 and folate deficiency, excessive alcohol consumption, syphilis, and risk factors for cardiovascular disease, such as atherosclerosis and diabetes. The results were required to be normal: complete blood count (CBC), thyroid-stimulating hormone (TSH), T3, T4, folic acid, vitamin B12, albumin, total cholesterol, HDL/LDL, triglycerides, alanine transaminase (ALT), aspartate transaminase (AST), gamma-glutamyl transferase (GGT), sodium, potassium, urea, creatinine, fasting glucose, VDRL, and ELISA anti-HIV test.

*(2) Brain MRI Scan*. All the patients were examined through brain MRI scans, analyzed by two experts MD. MRI based exclusion criteria were evidence of focal or lacunar ischemia, expansive brain tumors, and hydrocephalus. Changes related to normal aging, such as foci of rare nonspecific gliosis, were accepted. All participants had ischemic score Hachinski <7 (original) and <5 (modified by Loeb).

*(3) Neuropsychological Examination*. Next, a neuropsychological and functional activity battery was applied as inclusion and outcome criteria: IQCODE, Informant Questionnaire on Cognitive Decline in the Elderly [55]; B-ADL, Bayer Activities of Daily Living Scale [56].

*Memory Tests*. (1) MMSE, Minimental State Examination [57], an effective 11-question test used as a screening instrument to separate patients with cognitive impairment from those without it and (2) RBMT, the Rivermead Behavioural Memory Test [58], a brief test battery to assess everyday memory, with high level of ecological validity and good correlation with traditional episodic memory, were carried out [59, 60]. The RBMT consists of 12 subtests each of which addresses an important aspect of everyday memory function, mimicking daily life situations, recalling the first and last names; immediate and delayed recalling of a route, of a short story, and of a message (remembering to pick up an envelope and place it in a specific place); remembering to retrieve a personal belonging at the end of the examination and to ask for an appointment when an alarm sounds; immediate and delayed recalling of photographs of people, and nine questions about time and spatial orientation. (3) Logical memory (LM) I and II subtests of Wechsler Memory Scale [61], to measure encoding, retrieval, and logical memory ability were carried out. In LM I subtest, two short stories are presented and the examinee is asked to retell each one from memory immediately after hearing it. On the other hand, in LM II the delayed condition assesses long-term narrative memory with free recall and recognition tasks; (4) RAVLT, Rey Auditory-Verbal Learning Test [62–64], to evaluate short-term auditory-verbal memory, rate of learning, learning, and retrieval was carried out. Subjects repeat lists of 15 unrelated words over five different trials and then again after 30 minutes. From Wechsler Adult Intelligence Scale III [65], we applied two working memory subtests [66]: (1) letter-number sequencing test, where the participant is presented with a series of numbers and letters in random order and is instructed to repeat back letters and numbers combinations, first numbers in ascending order and then letters in alphabetical order [66] and (2) digit span, where the examinee repeats in direct and reverse order two series of three and two digits, respectively, read by the examiner. Executive function tests (frontal lobe tasks) were carried out. (1) Trail Making Test (TMT) A/B [67–70], to evaluate psychomotor speed, focus, visual search, mental flexibility, and sequencing, was carried out. In TMT-A, participants are asked to draw lines sequentially connecting 25 encircled numbers; task requirements are similar for TMT-B except for the fact that the person must differentiate between numbers and letters. The score represents the amount of time required, in seconds, to complete each task. (2) Verbal fluency tests, FAS and animal naming [71], to assess, respectively, the measure of total number of words generated in one minute for the letters F, A, and S (phonemic fluency) and of animal names (semantic fluency) were carried out; (3) Victoria Stroop Test [72] involves three trials. Three cards are presented in the same sequence and the examinee is instructed to read or call out the color name as quickly as possible. First, in the “word trial,” the subject reads words of color names (e.g., red and blue) printed in black ink; secondly, in the “color trial,” they identify colors (e.g., rectangles printed in red or blue). Finally, in the “color-word” response inhibition trial, they must name the color in which a word is presented, while ignoring the printed word.

All the scores were adjusted according to age, gender, and education level, and the tests were administered in accordance with the standard procedures.

### 2.2. Exclusion Criteria

The exclusion criteria are listed as follows: psychiatric disorders (except remitted depression ≥ 12 months) and alcohol and/or drug abuse, according to SCID-P [73], neurological conditions, severe uncontrolled organic disease, use of pacemaker, history of seizures, history of major head trauma, history of neurosurgery, and cerebral metallic artifacts.

### 2.3. TMS Procedures

Participants were randomly assigned in a double-blind condition to receive either active or sham rTMS. Randomization was performed through a random number generator (http://www.random.org) by a third-party investigator. Patients and rater were blinded to patients' treatment.

We used a high-speed magnetic stimulator (MagPro X100, MagVenture A/S, Farum, Denmark) with a figure-of-eight coil.

We used for the sham group a placebo coil, with a mechanical outline and sound level (click) identical to the active one. The placebo coil's magnetic shield provides a field reduction of approximately 90% [46, 74]. The motor threshold (MT) for each patient was determined by contraction of the right abductor of* pollicis brevis *muscle of the thumb, following the method described by Wassermann et al. [75].

rTMS was applied over the left DLPFC at the point located approximately 5 cm in a parasagittal plane parallel to the point of maximum stimulation of the short abductor of the thumb, with the lowest possible intensity in five of ten stimuli.

Subjects assigned to the active group received 10 Hz rTMS at 110% of MT, each train lasting 5 seconds, with 25-second intervals (2,000 pulses/day) for 10 consecutive weekdays. The sham group received the same protocol using a placebo coil.

At the end of the study period, after blinding was removed, the sham patients were given the option of receiving active rTMS treatment.

Security and side effects scales were assessed through a questionnaire as well as clinical evaluation, based on the most frequent adverse effects of TMS by The Safety of TMS Consensus Group [33].

### 2.4. Blind Condition

Patients and team raters were blinded to the assignment condition; however, for technical reasons, the clinicians who administered the rTMS were not. The rater was an experienced neuropsychologist, blinded to the treatment status and with no contact with the treatment team.

After completing the sessions, patients were asked what treatment they thought they received and why.

A lab researcher (C. G. M.) generated and concealed the random allocation sequence, and a secretary (S. L. F.) enrolled and assigned participants to interventions. The effectiveness of the blinding was assessed after the follow-up period.

### 2.5. Efficacy Variables

The primary outcome variable was the RBMT, for assessing everyday memory.

The secondary efficacy outcome variables were other neuropsychological domains assessments.

### 2.6. Statistical Analysis

Statistical analysis was performed by the SPSS v. 14 (Statistical Package for the Social Sciences, Chicago, IL, 2005). The Kolmogorov-Smirnov test was conducted to assess whether continuous variables followed a normal distribution. Statistical significance for all analyses was set to *α* = 5%.

Descriptive statistical analysis was performed for demographics: contingency tables for categorical variables (gender, comorbidity, marital status, and education level) and descriptive measures (mean and standard deviation) for continuous variables (age). The Fisher's exact test was used to verify the association of categorical variables. A Student's *t*-test was used to compare the mean of continuous normally distributed variables of both groups; the Mann-Whitney-Wilcoxon test was used when the variables did not follow a normal distribution. Two-way analysis of variance (ANOVA) for repeated measures compared group and time effect, as compared to the normal distribution of the data or residues. The blind control was evaluated by Cohen's kappa coefficient of agreement to assess patients' views of whether or not they belonged to a given group.

### 2.7. Flow Chart

Table 2 shows the overall structure of this study.

## 3. Results

### 3.1. Subjects

**Figure 1:**
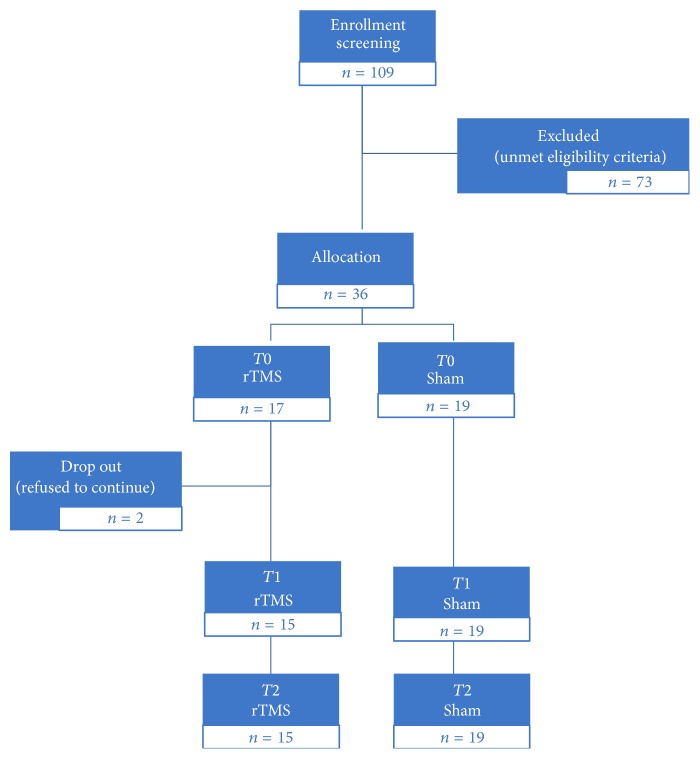
Flow diagram of referred and enrolled patients.

Out of 109 screened subjects, 73 did not fulfill the enrollment criteria. Among the 36 subjects left, 17 were randomly assigned to the active group and 19 to the sham group. In the active group, two drop-out subjects, after the first session, were excluded due to inability to follow the protocol. Therefore, 34 subjects entered the treatment phase (Figure 1). Among them, 31 were classified as a-MCI and three as nonamnesic-MCI (two in the sham group and one in the active group).

Causes of exclusion are listed in Table 3.

In the first step of the screening phase, no statistically significant difference was observed among the selected subjects (Table 4). There were no diagnostic cases of late life depression, nor present depression, excluded by various validated tests in preliminary evaluation, such as GDS, HAMD-17, and SCID DSM-IV.

Clinical and demographic characteristics were also similar in both groups, as seen in Table 5. At baseline, groups were homogeneous in terms of neuropsychological examination, except for digit span (*p* = 0.040).

### 3.2. Blind Integrity

An assessment of the effectiveness of the blinding revealed that most patients did not guess correctly, when asked to which group they believed they were assigned. The Kappa coefficient was equal to 0.190, which indicates a low correlation and blind integrity.

### 3.3. Tolerability and Safety

rTMS at 10 Hz with 110% of the MT was safe and well tolerated. A zero value presented in almost all cells of the side effects precludes any statistical analysis beyond a descriptive one. Side effects were mild and transient prevailing in the active group. However, a gradual reduction in side effects was observed throughout the sessions (see Table 6).

### 3.4. Outcome Variables

Four neuropsychological tests showed heterogeneous statistical improvement along time (Table 7).

**Figure 2:**
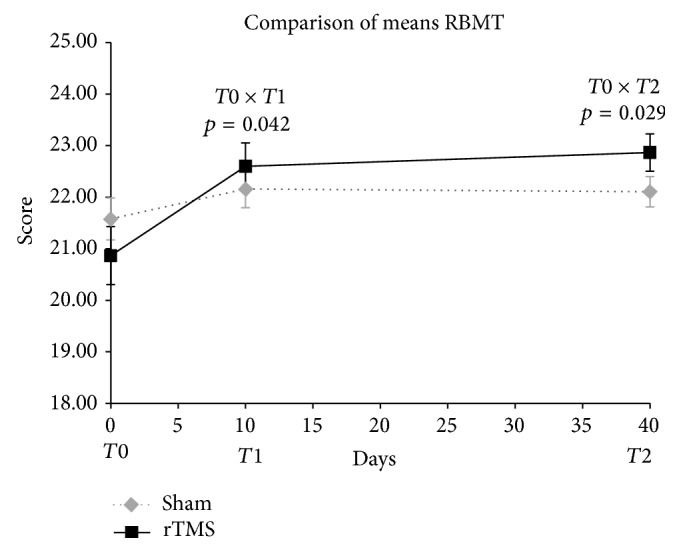
Comparison of RBMT means scores in T0, T1, and T2. Two-way ANOVA for repeated measures. Timing of procedures: T0: baseline cognitive assessment and 1st rTMS; T1: 10th rTMS session and 2nd cognitive assessment; T2: 30 days after T1 and 3rd cognitive assessment. Student's *t*-test for comparison of rTMS versus sham basal means, *p* = 0.292.

The primary outcome variable RBMT was statistically higher in the active group after the 10th session and after one-month follow-up (Figure 2).

**Figure 3:**
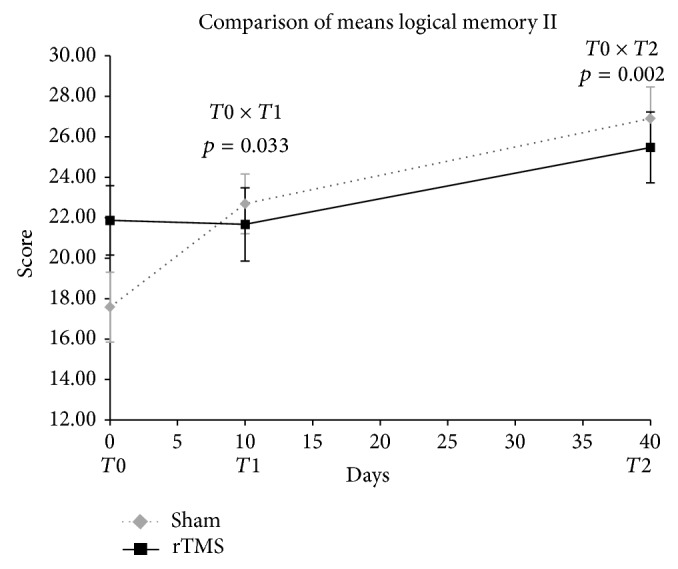
Comparison of logical memory II means scores in T0, T1, and T2. Two-way ANOVA for repeated measures. Timing of procedures: T0: baseline cognitive assessment and 1st rTMS; T1: 10th rTMS session and 2nd cognitive assessment; T2: 30 days after T1 and 3rd cognitive assessment. Student's *t*-test for comparison of rTMS versus sham basal means, *p* = 0.087.

Although final scores of the logical memory II were similar, initial values for the sham group indicate a significance favoring them (Figure 3).

**Figure 4:**
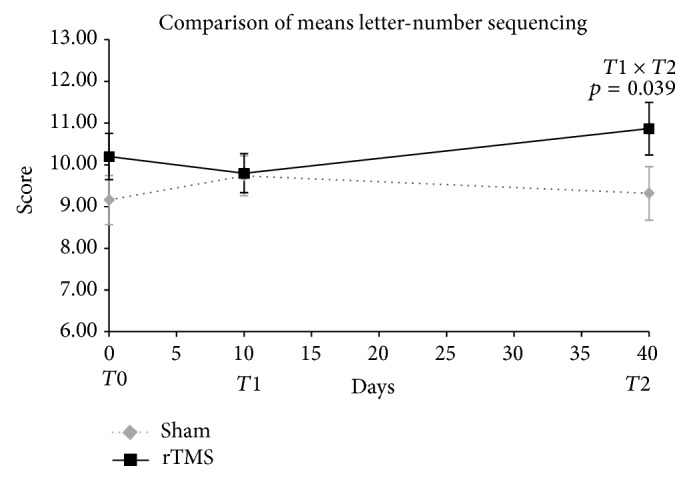
Comparison of letter-number sequencing means scores in T0, T1, and T2. Two-way ANOVA for repeated measures. Timing of procedures: T0: baseline cognitive assessment and 1st rTMS; T1: 10th rTMS session and 2nd cognitive assessment; T2: 30 days after T1 and 3rd cognitive assessment. Student's *t*-test for comparison of rTMS versus sham basal means, *p* = 0.211.

Figure 4 shows initial improvement in letter-number sequencing test for the sham group (T0-T1). Nevertheless, the gain for the active group at T2 did not show a significant difference at the end of follow-up (T2).

**Figure 5:**
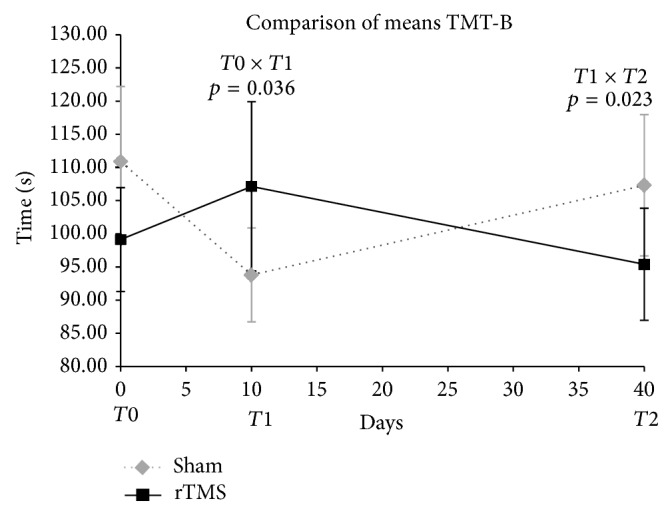
Comparison of Trail Making Test B means scores in T0, T1, and T2. Two-way ANOVA for repeated measures. Timing of procedures: T0: baseline cognitive assessment and 1st rTMS; T1: 10th rTMS session and 2nd cognitive assessment; T2: 30 days after T1 and 3rd cognitive assessment. Mann-Whitney-Wilcoxon test for comparison of rTMS versus sham basal means, *p* = 0.986.

In TMT-B, an initial improvement in the sham group was showed as well as, conversely, a later improvement in the active rTMS group (T1-T2). No definitive effect was shown in either group from basal to last evaluation (Figure 5).

**Figure 6:**
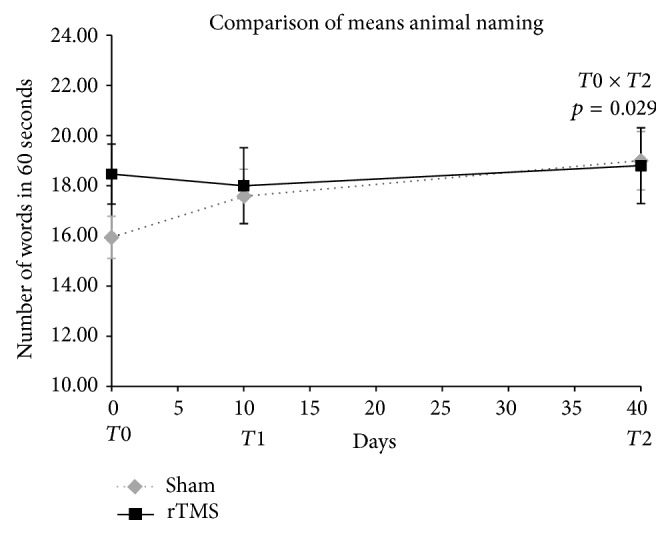
Comparison of semantic verbal fluency/animal naming means scores in T0, T1, and T2. Two-way ANOVA for repeated measures. Timing of procedures: T0: baseline cognitive assessment and 1st rTMS; T1: 10th rTMS session and 2nd cognitive assessment; T2: 30 days after T1 and 3rd cognitive assessment. Student's *t*-test for comparison of rTMS versus sham basal means, *p* = 0.081.

Transient improvement was observed in the sham group in verbal fluency/animal naming, at T2. However, the final scores were similar in both groups and quite heterogeneous in T0 (Figure 6).

## 4. Discussion

We report improvement in everyday memory after 10 sessions of HF rTMS, in a double-blind, randomized sham-controlled study. The duration of the improvement persisted at least for 30 days after the last rTMS session, assessed by the RBMT. This is the first randomized, controlled, double-blind study on early and late after-effects of rTMS on everyday memory of the elderly with MCI. This result suggests a sustained gain in episodic memory. The RBMT aids to identify compensatory strategies and to design specific neuropsychological rehabilitation programs. As the tasks mimic daily life situations, RMBT analyses individuals' tasks performances and how memory impairment affects everyday activities [76].

Nevertheless, others memory tests, logical memory (LM) II and letter-number sequencing (LNS) exhibited different outcomes. Sham group improvement in LM II is probably due a tendency to different baseline scores between both groups. A gain in the score of the active group in T2 is noted and can suggest a lag practice effect compared to that which may occur in sham group. In LNS test, we have an improvement of sham group at T1 and impairment at T2. Conversely, active group performance suggested a temporary deterioration soon after rTMS protocol (T1), followed by lag amelioration at T2.

Concerning the two frontal tasks, TMT-B and the verbal fluency test animal naming, the results showed some discrepancies. In TMT-B, there was an initial impairment in the active TMS group, followed by a great improvement after a month. Conversely, in animal naming test, the sham group had a gain and then impairment at the last evaluation, but the improvement of sham group may be due a tendency to statistical difference between baseline scores, which should require a larger sample to better define the result.

Anyway, this raises the possibility that the rTMS could have, at least in a short term, some negative effect on some performances. Even if most of the TMS findings show considerable variability, genetic factors can be argued. The presence of BDNF-Val66Met polymorphism could influence the protein synthesis, affecting cortical reactivity with decreased experience-dependent plasticity induced by rTMS. Thereby, this genetic variation in the normal population can produce significant differences in the after-effects of rTMS protocols [34, 43]. Koch et al. investigated the correlation between motor cortical plasticity (with TMS) and the levels of Ab, total tau (t-Tau), and phosphorylated tau detected in cerebrospinal fluid (CSF) of patients with AD. They identified that higher CSF t-Tau levels were associated with a stronger inhibition of the MEPs, suggesting that also CSF t-Tau modulates excitatory activity and may alter mechanisms of cortical plasticity. In one study of HF rTMS to bilateral PFC of patients with depression, Loo et al. [77] found an individual temporary deterioration in executive function/planning in the HF rTMS; two years later, the same group manifested a selective deterioration in the retention of verbal material [78].

One of the strengths of our study is its ecological validity. The patients recruited actively sought healthcare for memory disturbance in the community, through the media (radio and newspapers) and ads in the subway and buses, even by referral of fellow physicians or participants themselves.

Some peculiarities about rTMS efficacy in elderly populations are consistent with our data. It is well described that there is a better response to higher frequencies and intensity pulses rTMS, which should be explained by the greater prefrontal atrophy in the elderly. Due to cerebral atrophy, the distance from the skull to the PFC increases with age in greater proportion than the motor cortex [34, 79–82]. So, also, longer treatment protocols may be more effective [42, 83]. Moreover, patients tend to reach a greater improvement than healthy participants [50].

The duration of off-line rTMS after-effects in cognitive performance seems to indicate that longer trains induce longer-lasting and more robust effects, and rTMS parameters used in this study were consistent with those recommended on the induction of long-term cognitive effects (off-line paradigm) after more than one session of HF rTMS [30, 84].

Besides distant activations via neural pathways projections from the target of stimulation, the length of action of rTMS also depends on the rTMS “dose,” that is, the intensity of stimulation [37], which is directly related to the interindividual resting motor threshold.

Specific particularities influencing the interpretation of the results should be considered. First, due to the presence of a continuum of memory impairment from normal aging to MCI [85], the problem of high heterogeneity within our sample might be an important issue. Second, the “5 cm rule,” presents many limitations [86–88]. Third, we did not use different versions of RBMT, possibly introducing a bias although the practice effect is also present in all neuropsychological batteries. Finally, a selection bias may have occurred, due a diagnostic revision in a consensus meeting with the neuropsychology team. The initial goal was a sample of patients with a-MCI, that is, MCI subtype which is most susceptible to conversion to AD [1]. However, to keep the randomization, we maintained the three patients (9%) with nonamnesic MCI.

Interventional therapies studies for improving cognitive skills are of paramount importance and are likely to have a great impact on public health. The growing proportion of older people and the length of life increase through the world rapidly. Such issue requires the development of interventions to improve well-being, social engagement, and independence for ageing people [89].

There is a great interest in neuromodulation by rTMS due the persistence of after-effects induced by LTP mechanism [27, 30, 34, 37, 90–92]. LTP is an increase in the synaptic strength that could last for days or even weeks and months. Once induced and expressed, LTP is divided in two forms: early-LTP (E-LTP) and late-LTP (L- LTP). E-LTP is an increase in synaptic strength that persists for 30–60 minutes after induction, depending on modifications of existing proteins, for example, protein phosphorylation. L-LTP could last for hours, days, or even weeks and includes other mechanisms like changes in gene expression and the synthesis of proteins [30]. The duration of rTMS after-effect is proportional to the length of stimulation [84].

Most studies on healthy aging are focused on prevention. rTMS can be viewed as a tool for cognitive enhancement of the elderly with MCI, reversing or compensating cognitive deficits [4, 93] and improving quality of life. rTMS may interact synergistically with cognitive training to lead to even greater neurocognitive enhancement [93–95]. The elderly might benefit from cognitive rehabilitation with rTMS as an add-on instrument in cognitive training programs of a variety of neurological and cognitive disorders [96].

## 5. Conclusion

In conclusion, this study suggests that 10 consecutive sessions or HF rTMS to the left DLPFC at 10 Hz in the elderly with MCI selectively improve everyday memory. The improvement was sustained for at least a month. rTMS may be a promising useful tool for interventional single (or combined) therapy for individuals with MCI or with memory decline. Further research is necessary to replicate these findings with larger sample size and also to investigate rTMS combined with other cognitive training therapies.

## Figures and Tables

**Table 1:** Screening tests for MCI cut-off points.

Screening battery	Cut-off scores
MoCA Test^1^	≤24
CDR^2^	=0
GDS-15^3^	<5
HAMD-17^4^	<7
HAMA-14^5^	<8

^1^MoCA test: Montreal Cognitive Assessment test; ^2^CDR: Clinical Dementia Rating; ^3^GDS-15: 15-Item Geriatric Dementia Scale; ^4^HAMD-17: 17-Item Hamilton Depression Scale; ^5^HAMA-14: 14-Item Hamilton Anxiety Scale.

**Table 2:** Study structure timing.

(T-2) = screening part I
Clinical and demographic data
MoCA test^1^, CDR^2^, and GDS-15^3^
HAMD-17^4^ and HAMA-14^5^
Signed informed-consent forms

(T-1) = screening part II
IQCODE^6^ and B-ADL^7^
Lab blood sample analysis
Brain MRI/Hachinsky Ischemic Score
SCID DSM-IV^8^
Randomization

(T0) = 1st cognitive assessment battery^§^ (baseline)
1st rTMS session
Collateral effects scale

(T1) = 10th rTMS session
2nd cognitive assessment batery
Collateral effects scale

(T2) = one month after T1
3rd cognitive assessment battery
IQCODE and B-ADL

^1^MoCA test: Montreal Cognitive Assessment test; ^2^CDR: Clinical Dementia Rating; ^3^GDS-15: 15-Item Geriatric Dementia Scale; ^4^HAMD-17: 17-Item Hamilton Depression Scale; ^5^HAMA-14: 14-item Hamilton Anxiety Scale; ^6^IQCODE: Informant Questionnaire on Cognitive Decline in the Elderly; ^7^B-ADL: Bayer Activities of Daily Living Scale; ^8^SCID-DSM-IV: Structured Clinical Interview for DSM-IV Axis I Disorders-Diagnostic and Statistical Manual of Mental Disorders, fourth edition. ^§^Cognitive assessment battery: MMSE: Minimental State Examination; RBMT: Rivermead Behavioral Memory Test; WMS: Wechsler Memory Scale; WAIS: Wechsler Adult Intelligence Scale; RAVLT: Rey Auditory-Verbal Learning Test; Stroop: Stroop Color-Word Interference Test; Trail Making Test A/B.

**Table 3:** Causes of exclusion in the screening phase.

Excluded	*n*	Percentage
MoCA^1^ > 26	7	9.59%
Education level <4 years	2	2.74%
Depressive symptoms (GDS-15^2^ > 5)	25	34.25%
Effective bipolar disorder (SCID-DSM IV^3^)	7	9.59%
Anxiety	10	13.70%
Alcoholism	5	6.85%
Chronic benzodiazepine use	1	1.37%
Sleep disorders	4	5.48%
Epilepsy	4	5.48%
History of traumatic brain injury	2	2.74%
Cerebral MRI^4^ disorders	15	20.55%
Normal pressure hydrocephalus	2	2.74%
Lacunar infarct/ischemic stroke	8	10.96%
Frontoparietal meningioma	1	1.37%
Cerebellar cyst	1	1.37%
Neurocysticercosis	1	1.37%
Frontal granuloma	1	1.37%
Hemorrhagic lesion	1	1.37%
Frontal lobe atrophy	1	1.37%
Mild AD^5^	3	4.11%
Parkinson disease	3	4.11%
Frontotemporal dementia	1	1.37%

^1^MoCA: Montreal Cognitive Assessment; ^2^GDS-15: 15-items Geriatric Depression Scale; ^3^SCID: Structured Clinical Interview for Axis I Disorders-Diagnostic and Statistical Manual of Mental Disorders, fourth edition; ^4^MRI: magnetic resonance imaging; ^5^AD: Alzheimer's disease.

**Table 4:** Subjects screening: part I.

Test/scale	Active rTMS *n* = 15 (mean ± SD^∗^)	Sham *n* = 19 (mean ± SD)	*p*-value^∗∗^
MoCA^1^	24.5 ± 1.8	24.2 ± 2.3	0.605
GDS-15^2^	1.7 ± 1.7	1.4 ± 1.3	0.559
HAMD-17^3^	1.7 ± 2.1	1.5 ± 2.1	0.781
HAMA-14^4^	1.7 ± 1.1	1.4 ± 1.5	0.532

^∗^SD: standard deviation; ^∗∗^Student's* t*-test. ^1^MoCA: Montreal Cognitive Assessment; ^2^GDS-15: 15-item Geriatric Depression Scale; ^3^HAMD–17, 17-item Hamilton Depression Scale; ^4^HAMA–14, 14-item Hamilton Anxiety Scale.

**Table 5:** Demographic data.

Features	Active rTMS (*n* = 15)	Sham (*n* = 19)	*p*-value
Age, years (mean ± SD)	65.1 ± 3.5	65.2 ± 4.1	0.954^1^
Gender, *n* (%)			0.724^2^
Male	6 (40.0)	6 (31.6)	
Female	9 (60.0)	13 (68.4)	
Education level, years (mean ± SD)	15.1 ± 4.4	12.4 ± 4.7	0.094^1^
Marital status, *n* (%)			0.909^2^
Married	8 (53.3)	8 (42.1)	
Single	2 (13.3)	4 (21.1)	
Widow	3 (20.0)	4 (21.1)	
Divorced	2 (13.3)	3 (15.8)	
Residence			0.053^2^
Living alone, *n* (%)	1 (67)	7 (36.8)	
Living with family, *n* (%)	14 (93.3)	12 (63.2)	
Professional activities,* n* (%)			0.288^2^
Not retired	7 (46.7)	5 (26.3)	
Retired	8 (53.3)	14 (73.7)	
Physical activity, *n* (%) (≥twice a week, ≥1 year)	9 (60.0)	12 (63.2)	>0.999^2^
Comorbidities, *n *(%)			
Hypertension	9 (60.0)	5 (26.3)	0.080^2^
Diabetes mellitus	2 (10.5)	2 (13.3)	>0.999^2^
Dyslipidemia	9 (60.0)	9 (47.4)	0.510^2^
Thyroid disease	7 (46.7)	4 (21.1)	0.151^2^
Osteoporosis	3 (20.0)	5 (26.3)	>0.999^2^
Tobacco consumption	1 (6.7)	1 (5.3)	>0.999^2^
Neoplasia	1 (6.7)	2 (1.5)	>0.999^2^

SD: standard deviation; ^1^Student's* t*-test; ^2^Fisher's test.

**Table 6:** Side effects after rTMS sessions.

	# Sessions^∗^	1	5	10
Side effects^§^	Group	*n* (%)	*n* (%)	*n* (%)
Headache	Active rTMS	5 (33.3)	4 (26.7)	1 (5.3)
	Sham	5 (33.3)	0 (0)	0 (0)

Cervical pain	Active rTMS	0 (0)	0 (0)	0 (0)
	Sham	1 (5.3)	0 (0)	0 (0)

Scalp pain	Active rTMS	5 (33.3)	2 (13.3)	2 (13.3)
	Sham	1 (5.3)	1 (5.3)	0 (0)

Burning scalp	Active rTMS	0 (0)	0 (0)	0 (0)
	Sham	1 (5.3)	0 (0)	0 (0)

Concentration difficulties	Active rTMS	0 (0)	0 (0)	0 (0)
	Sham	0 (0)	0 (0)	0 (0)

^§^Symptoms related to rTMS application; ^∗^side effects after the 1st, 5th, and 10th rTMS sessions, respectively.

**Table 7:** Comparison of the statistically significant neuropsychological outcomes.

		T0		T1		T2		*p-*value^§^		
		Mean	SD	Mean	SD	Mean	SD	T0 × T1	T1 × T2	T0 × T2
RBMT	Active rTMS	20.87	2.10	22.60	1.68	22.87	1.36			
	Sham	21.58	1.77	22.16	1.57	22.11	1.29			
	Group effect							0.042^∗^	0.593	0.029^∗^

Logical memory II (delayed)	Active rTMS	21.87	6.40	21.67	6.79	25.47	6.56			
	Sham	17.58	7.50	22.68	6.44	26.89	6.81			
	Group effect							0.033^∗^	0.821	0.002^∗^

Letter-number sequencing test	Active rTMS	10.20	2.08	9.80	1.74	10.87	2.36			
	Sham	9.16	2.57	9.74	2.08	9.32	2.79			
	Group effect							0.130	0.039^∗^	0.489

Trail making test B	Active rTMS	99.13	29.26	107.13	47.87	95.40	31.58			
	Sham	110.89	49.31	93.79	30.84	107.32	46.45			
	Group effect							0.036^∗^	0.023^∗^	0.988

Verbal fluency/animal naming	Active rTMS	18.47	4.49	18.00	5.66	18.80	5.65			
	Sham	15.95	3.66	17.58	4.69	19.00	5.08			
	Group effect							0.095	0.613	0.029^∗^

^§^Analysis performed with two-way ANOVA for repeated measures (*p* > 0.05); ^∗^statistically significant group effect; SD: standard deviation; timing of procedures: T0: baseline cognitive assessment before 1st rTMS; T1: 2nd cognitive assessment (after 10th rTMS session); T2: 3rd cognitive assessment (30 days after T1).
